# Effects of amino acid substitutions in the VP2 B-C loop on antigenicity and pathogenicity of serotype Asia1 foot-and-mouth disease virus

**DOI:** 10.1186/1743-422X-9-191

**Published:** 2012-09-10

**Authors:** Mei Xue, Haiwei Wang, Wan Li, Guohui Zhou, Yabin Tu, Li Yu

**Affiliations:** 1 Division of Livestock Infectious Diseases, State Key Laboratory of Veterinary Biotechnology, Harbin Veterinary Research Institute, Chinese Academy of Agricultural Sciences, No. 427 Maduan Street, Harbin, 150001, P. R. China

**Keywords:** Foot-and-mouth disease virus, VP2, Antigenic variation, Replication ability, Virulence

## Abstract

**Background:**

Foot-and-mouth disease virus (FMDV) exhibits a high degree of antigenic variability. Studies of the antigenic diversity and determination of amino acid changes involved in this diversity are important to the design of broadly protective new vaccines. Although extensive studies have been carried out to explore the molecular basis of the antigenic variation of serotype O and serotype A FMDV, there are few reports on Asia1 serotype FMDV.

**Methods:**

Two serotype Asia1 viruses, Asia1/YS/CHA/05 and Asia1/1/YZ/CHA/06, which show differential reactivity to the neutralizing monoclonal antibody (nMAb) 1B4, were subjected to sequence comparison. Then a reverse genetics system was used to generate mutant versions of Asia1/YS/CHA/05 followed by comparative analysis of the antigenicity, growth property and pathogenicity in the suckling mice.

**Results:**

Three amino acid differences were observed when the structural protein coding sequences of Asia1/1/YZ/CHA/06 were compared to that of Asia1/YS/CHA/05. Site-directed mutagenesis and Immunofluorescence analysis showed that the amino acid substitution in the B-C loop of the VP2 protein at position 72 is responsible for the antigenic difference between the two Asia1 FMDV strains. Furthermore, alignment of the amino acid sequences of VP2 proteins from serotype Asia1 FMDV strains deposited in GenBank revealed that most of the serotype Asia1 FMDV strains contain an Asn residue at position 72 of VP2. Therefore, we constructed a mutant virus carrying an Asp-to-Asn substitution at position 72 and named it rD72N. Our analysis shows that the Asp-to-Asn substitution inhibited the ability of the rD72N virus to react with the MAb 1B4 in immunofluorescence and neutralization assays. In addition, this substitution decreased the growth rate of the virus in BHK-21 cells and decreased the virulence of the virus in suckling mice compared with the Asia1/YS/CHA/05 parental strain.

**Conclusions:**

These results suggest that variations in domains other than the hyper variable VP1 G-H loop (amino acid 140 to 160) are relevant to the antigenic diversity of FMDV. In addition, amino acid substitutions in the VP2 influenced replicative ability and virulence of the virus. Thus, special consideration should be given to the VP2 protein in research on structure-function relationships and in the development of an FMDV vaccine.

## Background

Foot-and-mouth disease (FMD) is a highly infectious and economically important disease of cloven-hoofed animals. The causative agent, foot-and-mouth disease virus (FMDV), is a small, non-enveloped virus that belongs to the *Aphthovirus* genus of the *Picornaviridae* family [1]. Like other picornaviruses, FMDV possesses a single-stranded positive-sense RNA genome of approximately 8,500 nucleotides that are encapsidated in an icosahedral capsid made of 60 copies each of four proteins: VP1, VP2, VP3 and VP4. Seven serotypes (A, O, C, Asia1, and South African Territories 1, 2, and 3) have been identified serologically, and multiple subtypes occur within each serotype [2]. The virus presents tremendous antigenic variability, which has been extensively characterized in the field [3-5].

Although major antigenic sites have been described in VP1 (residues 140 to 160 and the C terminus) [5-7], the use of monoclonal antibodies (MAb) to select neutralization-resistant mutant viruses has demonstrated the presence of other neutralization and non-neutralizable sites in other proteins of the virus particle [5,7-9]. In FMDV serotypes O, A and C, antigenic sites in VP2 were shown to be immunologically important [10-12], and mutations within these sites were able to affect antigenicity of these viruses. In addition, crystallographic data [13] and studies utilizing neutralization-resistant variants have shown that amino acids 70-80 of the VP2 B-C loop are located in close proximity to the VP1 G-H loop [14]. Indeed, at least one epitope in the VP2 B-C loop has been described for serotype Asia1 FMDV [4].

Serotype Asia1 FMDV has circulated widely in Asia after first being detected in samples collected in India between 1951 and 1952 and in Pakistan in 1954 [15]. In China, serotype Asia1 FMDV was first found in the Yunnan province in 1958 [16], and, at that time, this type of FMDV was confined to the YNBS lineage. In 2005, Jiangsu lineage serotype Asia1 FMDV Asia1/JS/CHA/05 [GenBank: EF149009], which was first isolated in India (Asia1/IND/18/80) [GenBank: DQ121116] (Asia1/IND/15/81) [GenBank: DQ121117], caused outbreaks in cattle in more than 15 areas of China and resulted in severe economic losses [17,18]. Interestingly, this serotype was subsequently isolated from pigs in 2006 [GenBank: FJ906802]. However, this viral lineage is poorly transmitted between pigs by the classical respiratory and digestive pathways, and it causes limited death in pigs. The present study examined two isolates: Asia1/YS/CHA/05, which was isolated from cows, and Asia1/1/YZ/CHA/06, which was isolated from pigs. These isolates showed different reactivity to the serotype Asia1 FMDV-specific monoclonal antibody 1B4. The Asia1/YS/CHA/05 virus reacted with MAb 1B4 in an indirect immunofluorescence assay (IFA) and a virus neutralization test (VNT), but the Asia1/1/YZ/CHA/06 virus did not react with MAb 1B4. We investigated the molecular basis for the differential reactivity of these two viruses against MAb 1B4 using a reverse genetics system. Our results demonstrate that a single amino acid variation at position 72 of the VP2 protein confers the antigenic difference between these two Asia1 FMDV isolates. Moreover, this amino acid substitution at position 72 in the B–C loop of VP2 affects the replicative ability and virulence of the virus.

## Results

### Production of MAb against Asia1/YS/CHA/05

Mouse spleen cells immunized with the inactivated Asia1/YS/CHA/05 FMDV were fused with SP2/0 myeloma cells to generate MAb 1B4. Isotope determination exhibited that MAb 1B4 was of the IgG1/κ-type. Virus micro-neutralization tests (VNT) showed that MAb 1B4 neutralized the Asia1/YS/CHA/05 virus with a neutralization titer of 1:64. However, MAb 1B4 failed to recognize the denatured FMDV protein in a western blot (data not shown), which suggests that the epitope recognized by MAb 1B4 is conformation dependent.

### Virus originating in pigs react weakly with MAb 1B4 in an immunofluorescence assay

**Figure 1:**
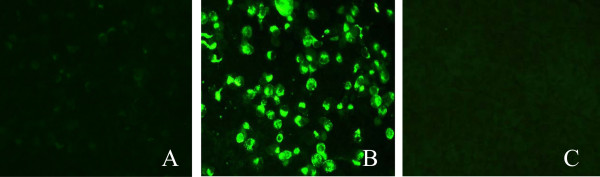
**Reactivity of Asia1/YS/CHA/05 and Asia1/1/YZ/CHA/06 with MAb 1B4 in an indirect immunofluorescence assay.** (**A**) Asia1/1/YZ/CHA/06-infected BHK-21 cells, **(B)** Asia1/YS/CHA/05-infected BHK-21 cells and (**C**) normal BHK-21 cells as a negative control.

Asia1/YS/CHA/05 was isolated from cattle during an important epizootic wave in China in 2005, and Asia1/1/YZ/CHA/06 was isolated from pigs in 2006. We performed an indirect immunofluorescence assay to test the reactivity of MAb 1B4 against these two FMDV strains. The results show that MAb 1B4 reacted very weakly with the Asia1/1/YZ/CHA/06 strain from pigs (Figure 1A), but it had strong reactivity against the Asia1/YS/CHA/05 strain from cows (Figure 1B).

### Sequence analysis of the capsid of Asia1/1/YZ/CHA/06 from pigs

In light of the significantly altered antigenic properties of Asia1/1/YZ/CHA/06 to MAb 1B4, we sequenced the structural protein-coding region of Asia1/1/YZ/CHA/06 to determine whether the antigenic alteration could be ascribed to sequence variation. Compared with Asia1/YS/CHA/05, three amino acid substitutions were found in the P1 coding region of Asia1/1/YZ/CHA/06, including a Ser-to-Asn substitution at position 154 (S154N) in VP1, an Asp-to-Gly substitution at position 72 (D72G) in VP2 and a Val-to-Ile substitution at position 107 (V107I) in VP2. Table 1 shows the amino acid substitutions that were different between the two viruses.

**Table 1:** Differences in amino acid residues in the P1 region between the porcine Asia1/1/YZ/CHA/06 FMDV and the bovine Asia1/YS/CHA/05 FMDV

**Strain**	**Host**	**Amino acid substitution**		
		**VP1/154**	**VP2/72**	**VP2/107**
Asia1/YS/CHA/05	bovine	Ser	Asp	Val
Asia1/1/YZ/CHA/06	pig	Asn	Gly	Ile

**Figure 2:**
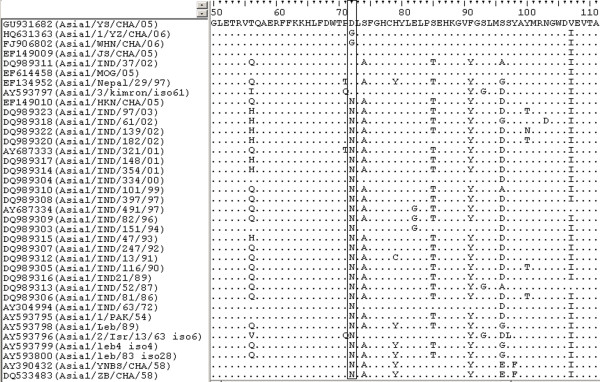
**Comparison of the VP2 amino acid sequence between Asia1 FMDV strains.** The amino acids at position 72 are boxed.

To investigate the origin of the mutated amino acid residues in the VP2 coding region of the porcine Asia1/1/YZ/CHA/06 virus, we aligned the Asia1 FMDV VP2 coding regions available in GenBank. Our results showed that there are three amino acid differences at position 72 of VP2 (Figure 2). In addition, the majority of Asia1 FMDV strains contain an Asn residue. Six strains (i.e., Asia1/YS/CHA/05, Asia1/JS/CHA/05 [GenBank: EF149009], Asia1/MOG/05 [GenBank: EF614458], Asia1/IND/37/02 [GenBank: DQ989311], Asia1/Nepal/29/97 [GenBank: EF134952] and Asia1/3/kimron/iso61 [GenBank: AY593797] had an Asp residue at position 72 of the VP2 protein. Importantly, Asia1/WHN/CHA/06 [GenBank: FJ906802], which is another virus that originated in pigs, also contains a Gly substitution at the same position as Asia1/1/YZ/CHA/06. These data show that the Asn residue at position 72 of VP2 is highly conserved among most Asia1 FMDV strains. However, Asn was replaced by Asp at position 72 of VP2 in the virus originating from the Indian 1980/1981 epidemics and the outbreaks in the cattle populations of China in 2005. In addition, Asn was replaced by Gly in the outbreak in pigs in 2006.

### Rescue of the site-directed mutants of Asia1/YS/CHA/05

**Figure 3:**
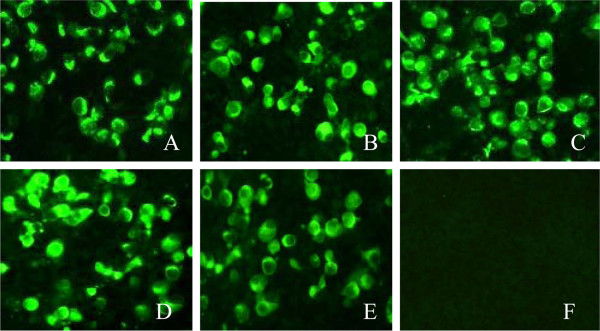
**Reactivity of the mutant viruses with MAb 4B2 by indirect immunofluorescence.** (**A**) rS154N-infected BHK-21 cells, (**B**) rD72G-infected BHK-21 cells, (**C**) rD72N-infected BHK-21 cells, (**D**) rV107I-infected BHK-21 cells, (**E**) Asia1/YS/CHA/05-infected BHK-21 cells as a positive control and (**F**) normal BHK-21 cells as a negative control.

Comparative analysis of the deduced amino acid sequences in Asia1/YS/CHA/05 and Asia1/1/YZ/CHA/06 shows that there were three amino acid substitutions in the P1 coding region of Asia1/YS/CHA/05 (Table 1). Thus, it was reasonable to assume that one of these substitutions contributed to the antigenic difference between these two viruses. To verify this assumption, we used an infectious clone of Asia1/YS/CHA/05 [6] as a backbone to generate three plasmids containing each of the mutations S154N, D72G and V107I described above. In addition, because most Asia1 FMDV isolates contain an Asn residue at position 72 of VP2, we also constructed a mutated pAsi plasmid with an Asp-to-Asn substitution at the same position of VP2. Infectious viruses were produced from these mutated cDNAs by *in vitro* transcription followed by transfection of BHK-21 cells. Viruses derived from the mutated plasmids were named rS154N, rD72G, rV107I and rD72N. The entire sequence of the four mutant viruses were determined by RT-PCR and sequencing, and no additional nucleotide changes were observed in cDNAs of the four mutant viruses. The antigenicity of the four mutant viruses was subsequently tested with an indirect immunofluorescence assay using the serotype-independent MAb 4B2. The results show that all four mutant viruses can specifically react with MAb 4B2 at the same level as the Asia1/YS/CHA/05 parental virus (Figure 3), which provides more evidence that the mutant viruses were rescued.

### Asp72 is a crucial residue of the 1B4 epitope in VP2

**Figure 4:**
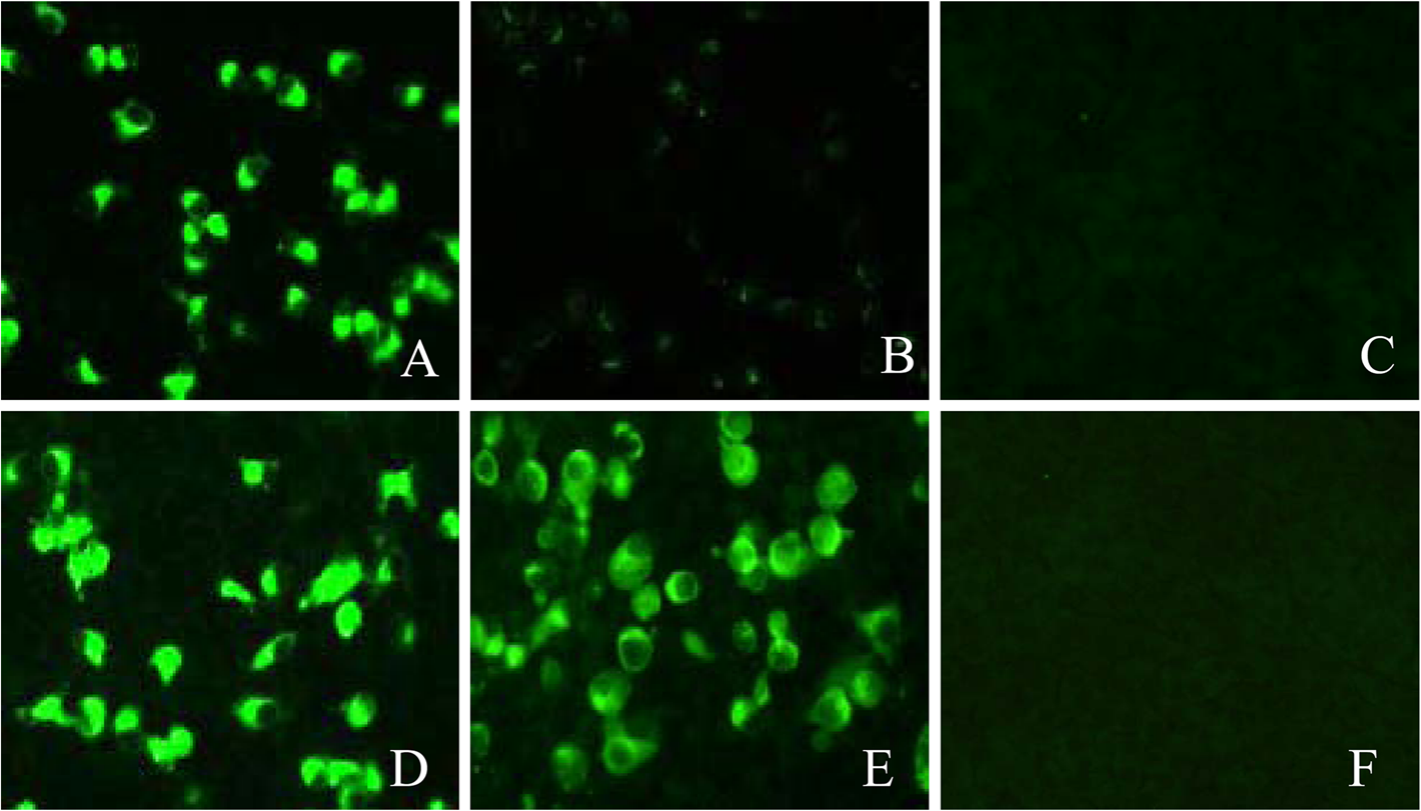
**Reactivity of mutant viruses with MAb 1B4 by indirect immunofluorescence.** (**A**) rS154N-infected BHK-21 cells, (**B**) rD72G-infected BHK-21 cells, (**C**) rD72N-infected BHK-21 cells, (**D**) rV107I-infected BHK-21 cells, (**E**) Asia1/YS/CHA/05-infected BHK-21 cells as a positive control and (**F**) normal BHK-21 cells as a negative control.

To assess the impact of the amino acid changes on the antigenicity of the Asia1/YS/CHA/05 parental virus, the reactivates of the four site-directed mutant viruses were tested against MAb 1B4 using an indirect immunofluorescence assay. Our results show that two of the mutant viruses, rS154N and rV107I, reacted equally well with MAb 1B4 as the Asia1/YS/CHA/05 parental virus (Figure 4A and Figure 4D), whereas the rD72G mutant virus reacted poorly with this MAb (Figure 4B), and the rD72N mutant virus did not react with MAb 1B4 (Figure 4C). These results demonstrate that an Asp residue at position 72 of the VP2 protein is crucial for recognition of the MAb 1B4 epitope. The isolate Asia1/1/YZ/CHA/06 from pigs with an Asp-to-Gly substitution at this position showed minor reactivity with MAb 1B4. However, the S154N substitution in VP1 and the V107I substitution in VP2 had no effect on the epitope recognized by 1B4, indicating that these residues were not related to the epitope recognized by MAb 1B4.

### Mutant viruses rD72G and rD72N escape from neutralization by 1B4

We performed virus neutralization tests to detect the effects of site-directed mutations on the neutralizing ability of MAb 1B4. The Asia1/YS/CHA/05 and Asia1/1/YZ/CHA/06 parental viruses were used as controls. The results showed that the Asia1/YS/CHA/05 could be neutralized by MAb 1B4 with a neutralization titer of 1:64, but the porcine Asia1/1/YZ/CHA/06 strain completely escaped neutralization by 1B4. For the mutant viruses, the V107I and S154N mutations had no effect on the neutralizing ability of MAb 1B4, whereas the rD72G and rD72N mutants completely escaped neutralization by the 1B4. These results were in agreement with those of the immunofluorescence assay (Figure 4), which further supports the notion that Asp72 is a crucial residue in the VP2 epitope neutralized by 1B4. Indeed, the viral epitope that could be neutralized by 1B4 disappeared when the Asp72 residue was substituted with Gly or Asn. These results showed that a single amino acid substitution in VP2 of Asia1 FMDV can have a dramatic effect on the neutralizing activity of the MAb 1B4.

### Replicative ability of the Asp72-mutant viruses

**Figure 5:**
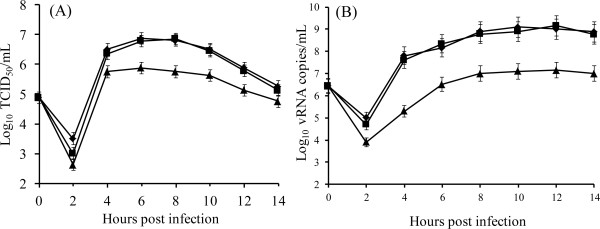
**Growth kinetics of rD72G and rD72N mutant viruses.** BHK-21 cells were infected and harvested at different times post infection. (**A**) Single-step growth curves obtained by titration of virus present at each time. Samples were taken in triplicates and standard deviations are shown in the graphic. (**B**) RNA production curves were obtained by real time RT-PCR assay from Trizol resuspended monolayers. The mean viral load generated by real time RT-PCR is expressed as log_10_ vRNA copies/ml of virus transport medium. Samples were taken in triplicates and standard deviations are shown in the graphic. (◆) FMDV Asia1/YS/CHA/05,(■) rD72G and (▴) rD72N.

We constructed single-step growth curves of virus production in BHK-21 cells to test whether the amino acid substitutions at position 72 of VP2 influenced the ability of the viruses to replicate in cell culture. Viral titers recovered at different times post infection were compared with those obtained with the parental Asia1/YS/CHA/05 (Figure 5A). In the cells infected with the parental virus and the rD72G mutant virus, viral titers increased by 4 hpi, reaching a maximum titer of approximately 10^7^ TCID_50_/mL at 6 hpi. After reaching this peak, titers gradually declined to 10^5^ TCID_50_/mL by the final sample collection time at 14 hpi. Although productive viral replication also occurred in rD72N-infected cells, viral titers were lower than those of parental virus and the rD72G mutant virus (Figure 5A). The trends in the viral growth curves were similar to those observed in nucleic acid copy numbers quantified by real-time RT-PCR (Figure 5B). BHK-21 cells infected with the parental virus and the rD72G mutant virus had increased viral nucleic acid copy numbers from 4 hpi, reaching a maximum after 8 hpi. In contrast, viral nucleic acid copy numbers in the cells infected with the rD72N remained low throughout the time course of the experiment. These data confirmed that the rD72N mutant virus replicates to lower titers in BHK-21 cells than does the parental virus and the rD72G mutant virus. These results showed that the Asp-to-Asn substitution at position 72 affected the replicative ability of the Asia1/YS/CHA/05 virus.

### Mutant virus rD72N is less virulent in suckling mice

To determine whether the substitutions in VP2 were related to virulence *in vivo*, 10-fold series dilutions of the rD72G and rD72N mutant viruses and the Asia1/YS/CHA/05 parental strain were inoculated into 3-day-old BALB/c suckling mice for evaluation of the 50% lethal dose (LD_50_). The results showed that the rD72G mutant virus induced disease in suckling mice with similar severity as the parent strain, and both viruses had similar LD_50_ titers (1.4 × 10^-8^ and 1.5 × 10^-8^, respectively). The rD72N mutant virus, however, was 10-fold less virulent in suckling mice, and the LD_50_ titer was 1.2 × 10^-7^. The experiment was repeated for three times, and the results were very consistent upon repeated experiments. These results indicated that an Asp-to-Asn, but not an Asp-to-Gly, substitution at position 72 of VP2 had an effect on virulence in mice. This was consistent with the *in vitro* replication ability of these mutant viruses in BHK-21 cells, suggesting that there is a link between growth characteristics and *in vivo* virulence.

## Discussion

Due to the error-prone nature of FMDV′s RNA-dependent RNA polymerase and high replication rate *in vivo*, FMDV exhibits a high degree of antigenic variability [19,20]. Studies of the antigenic diversity and determination of amino acid changes involved in this diversity are important to the design of broadly protective new vaccines [7]. Although extensive studies have been carried out to examine the epitopes in the viral capsids of serotype O [8,10,21] and serotype A FMDV [11,22,23], there are few reports on Asia1 serotype FMDV [24]. In this study, we explored the molecular basis of the antigenic difference between two viruses, Asia1/YS/CHA/05 from cows and Asia1/1/YZ/CHA/06 from pigs, which react differently against MAb 1B4. We detected three amino acid substitutions in the P1 coding region of Asia1/YS/CHA/05 by sequencing and alignment of these two viruses, including a Ser-to-Asn substitution at position 154 (S154N) in VP1, an Asp-to-Gly substitution at position 72 (D72G) in VP2 and a Val-to-Ile substitution at position 107 (V107I) in VP2 (Table 1). We expected that the S154N substitution in the VP1 G-H loop would be related to the variation in 1B4 recognition; however, single amino-acid substitutions based on an FMDV reverse genetics system revealed that the D72G substitution in VP2 contributed to this antigenic variation. Moreover, we found that the virus containing an Asp-to-Asn substitution (D72N) had a reduced ability to replicate in BHK-21 cells and was 10-fold less virulent in suckling mice. These results provide evidence that amino acid substitutions in the VP2 B-C loop of serotype Asia1 FMDV not only mediate significant antigenic diversity in the field but also alter the replicative ability and pathogenicity of the virus.

X-ray crystallographic data have shown that the B-C loop of VP2 lies in the vicinity of the G-H loop of VP1 [25]. In addition, amino acid changes in the B-C loop of VP2 in type O [10] and type A [11,23] FMDV have been shown to contribute to an immunodominant site in VP1. In this study, an Asp-to-Gly substitution or an Asp-to-Asn substitution at position 72, which comprises part of the B-C loop of VP2, inhibited the ability of the Asia1 FMDV strain to react with MAb 1B4. Thus, the Asp-to-Gly or Asp-to-Asn substitutions at position 72 of VP2 may affect the conformation and/or orientation of the VP1 G-H loop, which could impact the ability of MAb 1B4 to recognize its epitope.

Interestingly, two closely related viruses from type A22, which showed different behavior in cell attachment assays and had different host range properties, did not have any differences in the VP1 coding region; however, they did have three amino acid substitutions in VP2. One of these substitutions (Glu82 to Gly) on the surface of VP2 perturbed the structure of the VP1 G-H loop, which determined the cell-binding properties of the variants [26]. This report adds support to the idea that VP2 is important for the cell tropism of the virus. The natural hosts of most serotype Asia1 FMDV strains are cattle, and there is only one report of pigs infected with Asia1 serotype FMDV (i.e., IND17/91 [GenBank: AF390682]). Here, the Asia1/1/YZ/CHA/06 virus from pigs and ano-ther porcine isolate, Asia1/WHN/CHA/06 [GenBank: FJ906802], shared the same Asp-to-Gly substitution at position 72 of VP2. The loss of the aspartic acid side chain on the surface of VP2 in the porcine variants of Asia1 FMDV may perturb the G-H loop of VP1. Because a key determinant of the cellular tropism of FMDV is also located in the G-H loop of VP1 [27,28], we cannot exclude that the Asp-to-Gly substitution changed the host tropism of viral isolates from a similar lineage, which may explain the difference in host tropism of Asia1/YS/CHA/05 and Asia1/1/YZ/CHA/06. Further investigations are needed to elucidate the effects of these amino acid changes on FMDV infection of various species.

The D72N mutation decreased the growth rate of Asia1/YS/CHA/05 in BHK-21 cells. The results of *in vitro* growth analysis were consistent with those of *in vivo* virulence experiments, which suggest that the decreased growth activity of the virus caused by the D72N mutation may contribute to the decreased virulence of the rD72N mutant. A single Asp-to-Asn substitution at position 72 of VP2 may influence the structure of the virus capsid, which would account for the low replication ability and reduced virulence of the virus. Further studies are needed to clarify the mechanisms of these changes.

## Conclusions

In summary, we explored the molecular basis of differential reactivity of two closely related Asia1 serotype FMDV strains to MAb 1B4 by site-directed mutagenesis using a reverse genetics system. We found that a single amino acid substitution at position 72 in the B-C loop of VP2 resulted in variation in the epitope recognized by MAb 1B4 and escape from 1B4-mediated neutralization. Our results suggest that variations in domains other than the hyper variable VP1 G-H loop (amino acid 140 to 160) are relevant to the antigenic diversity of FMDV. In addition, amino acid substitutions in VP2 influenced the replicative ability and virulence of the virus. Thus, special consideration should be given to the VP2 protein in research on structure-function relationships and in the development of an FMDV vaccine.

## Materials and methods

### Ethics statement

Care of laboratory animals and animal experimentation were performed in accordance with animal ethics guidelines and approved protocols. All animal studies were approved by the Animal Ethics Committee of Harbin Veterinary Research Institute and by the Animal Ethics Committee of Heilongjiang Province (SYXK (H) 2006-032).

### Viruses, cells and plasmids

The Asia1/YS/CHA/05 FMDV isolate [GenBank: GU931682] was isolated during the pandemic in China in 2005 [17,29], and the Asia1/1/YZ/CHA/06 FMDV isolate [GenBank: HQ631363] was isolated from enzootic of pigs in 2006 [GenBank: FJ906802]. The baby hamster kidney cell line BHK-21 was maintained in Dulbecco′s modified Eagle′s medium supplemented with 10% fetal calf serum (Gibco-BRL, Grand Island, NY, USA). Viruses were propagated in BHK-21 cells. Plasmid pAsi, an infectious clone of Asia1/YS/CHA/05 FMDV, has been previously described [30].

### Monoclonal antibodies

Six-week-old female BALB/c mice were subcutaneously immunized with 100 μg of the inactivated and purified Asia1/YS/CHA/05 FMDV antigen emulsified in an equal volume of adjuvant VG206 (SEPTIC, France). Two boosters of the adjuvant-emulsified antigen were given at 2-week intervals. Two weeks after the third immunization, the mice were intraperitoneally boosted with 100 μg of antigen without adjuvant. Three days later, spleen cells were collected to fuse with SP2/0 myeloma cells using 50% (wt/vol) polyethylene glycol and 10% dimethyl sulfoxide (DMSO) (vol/vol) (Sigma, St. Louis, MO, USA). Hybridomas were screened by an enzyme-linked immunosorbent assay (ELISA) and verified by an indirect immunofluorescence assay (IFA) and a western blot. The MAb-producing hybridoma was cloned three times by limiting dilution of the cells. Antibody subtype identification was performed using the SBA Clonotyping^TM^ System/HRP Kit (Southern Biotech, Birmingham, AL, USA). The FMDV serotype-independent MAb 4B2 has been previously described [31].

### Site-directed mutagenesis and construction of recombinant plasmids

Plasmid pPM was generated by digesting plasmid pAsi with PstI and MluI to generate a fragment containing the VP1 gene. This fragment was ligated with plasmid pOK12, which had previously been digested with PstI and MluI. Plasmid pNP was generated by digesting pAsi with NdeI and PstI to generate a fragment containing the VP2 gene. This fragment was ligated with pOK12, which had previously been digested with NdeI and PstI. The plasmid pPM and pNP were used as templates for site-directed mutagenesis and the amplified DNA were digested with restriction enzyme DpnI according to the reference previously reported with minor modifications [32]. The primers used to introduce the specific mutation shown in Table 2. For PCR, the 50 μL reaction mixture was comprised of 10 μL of 5 × Prime STAR buffer, 4 μL of 2.5 mM deoxy ribonucleotide triphosphates (dNTP), 2 μL of forward primer, 2 μL of reverse primer, 1 μL of Prime STAR HS DNA polymerase, 30.5 μL of sterile ddH_2_O and 0.5 μL of pPM or pNP. The PCR protocol consisted of 4 min of pre-denaturation at 94°C; 18 cycles of 94°C for 1 min, 52°C for 30 sec and 68°C for 7 min; and a final extension at 68°C for 10 min. The PCR products were extracted with one volume of Tris-EDTA saturated (pH 4.5) phenol/chloroform/isoamyl alcohol (25:24:1) and precipitated with 1/10 volume 3 M sodium acetate (pH 5.2) and 2.5 volumes of anhydrous alcohol. The precipitated products were washed with 70% alcohol and digested with DpnI. After digestion, the mutant plasmids were transformed into *E. coli* DH5α cells and grown overnight at 37°C in the presence of Kanamycin. The resulting plasmid, designated pPM-N, was digested with PstI and MluI and ligated into pAsi, which had been digested with the same enzymes. The other constructed plasmids, designated pNP-G, pNP-N and pNP-I, were digested with NdeI and PstI and ligated into pAsi, which had been digested with the same enzymes. The final products were designated as pAsiPM-N, pAsiNP-G, pAsiNP-N and pAsiNP-I and verified by DNA sequencing.

**Table 2:** Oligonucleotide primers used for site-directed mutagenesis and sequencing

**Primer**	**Position**^**c**^	**Sequence**^**d**^	**Orientation**
154F^a^	3704-3740	5'-CCCTCGCACGCAGAGTG**AAC**AACCGGCTGCCCACTTC-3'	Sense
154R^a^	3704-3740	5'-GAAGTGGGCAGCCGGTT**GTT**CACTCTGCGTGCGAGGG-3'	Antisense
72GF^a^	2148-2181	5'-GTTTGACTGGACGCC**GGG**TTTGTCATTTGGACAC-3'	Sense
72GR^a^	2148-2181	5'-GTGTCCAAATGACAAA**CCC**GGCGTCCAGTCAAAC-3'	Antisense
72NF^a^	2148-2181	5'-GTTTGACTGGACGCCG**AAT**TTGTCATTTGGACAC-3'	Sense
72NR^a^	2148-2181	5'-GTGTCCAAATGACAA**ATT**CGGCGTCCAGTCAAAC-3'	Antisense
107F^a^	2253-2287	5'-GAGGAACGGGTGGGAC**ATT**GAGGTGACCGCTGTTG-3'	Sense
107R^a^	2253-2287	5'-CAACAGCGGTCACCTC**AAT**GTCCCACCCGTTCCTC-3'	Antisense
S1F^b^	207-227	5'-AACAGCTATGACCATGATTAC-3'	Sense
S1R^b^	2677-2697	5'-GTTAGACACTTTCCCGTAGAC-3'	Antisense
S2R^b^	4000-4019	5'-GACATGTCCTCCTGCATCTG-3'	Antisense

^a^ Primers for the construction of mutant viruses.

^b^ Primers for sequencing.

^c^ The position of primers was determined according to the sequence of pAsi.

^d^ The mutated codons are bolded and underlined.

### Generation of rescued viruses from cDNA clones

The mutant-containing plasmids were linearized with the restriction endonuclease EcoRV and used as templates for RNA synthesis with the RiboMAX Large Scale RNA Production Systems-T7 kit (Promega, Madison, WI, USA) according to the manufacturer′s instructions. After transcription, reaction mixtures were treated with 1 U RQ1 DNase per μg RNA (Promega, Madison, WI, USA). BHK-21 cells were Transfected with 5 μg of *in vitro* transcribed RNA using the Effectene® Transfection Reagent (Qiagen, Valencia, CA, USA). Supernatants from the Transfected cells were used to infect fresh BHK-21 cell monolayers. After a 48 h incubation at 37°C, viruses were harvested by three cycles of freeze-thawing and were passages three more times on fresh BHK-21 cell monolayers. Rescued viruses were examined by an immunofluorescence assay using MAb 4B2 and subjected to reverse transcriptase PCR (RT-PCR) for sequencing to confirm the introduced amino acid substitutions and the absence of other changes.

### Immunofluorescence analysis

BHK-21 cells were infected with the rescued viruses and the parent virus at a MOI of 5.0 in 96-well plates, fixed with ice-cold anhydrous ethanol 8 h post-infection for 15 min at 4°C and air dried. Fifty micro liters of MAb 4B2 or MAb 1B4 (1:200 dilution in PBS) was added to each well and incubated for 1 h at 37°C. After washing with PBS, 50 μL of FITC-conjugated goat anti-mouse IgG (1:200 dilution, Sigma, St. Louis, MO, USA) was added to each well and incubated for 1 h at 37°C. Plates were then washed three times with PBS and imaged with an Olympus microscope connected to a Leica DFC490 digital color camera.

### RNA isolation, RT-PCR, and nucleotide sequence analysis

Genomic RNA from the rescued viruses was extracted using TRIzol LS (Invitrogen, Carlsbad, CA, USA) according to the manufacturer’s instructions. The RNA was reverse transcribed into cDNA using Oligo-dT (15 T) as a primer, and the PCR-amplified products were subjected to nucleotide sequencing. The full-length genomic sequences of the mutant viruses were analyzed to confirm the introduced amino acid substitutions and the absence of other changes.

### Micro-neutralization assay

The micro-neutralization assay was performed according to the Office International des Epizooties (OIE) manual (http://www.oie.int/eng/normes/MMANUAL/2008/pdf/2.01.05_FMD.pdf). Briefly, two-fold serial dilutions of heat-inactivated ascitic fluid were incubated with 50 μL of 200 TCID_50_ units of FMDV for 1 h at 37°C in a 96-well micro plate. Approximately 10^4^ BHK-21 cells in 100 μL of media were seeded per well, and the cells were incubated with virus and Ascites for five days prior to being examined for a cytopathic effect. The assay was independently performed three times, and the neutralizing titer (NT_50_) was calculated using the Kärber method.

### Growth analysis of recombinant viruses in cell culture

To monitor virus replication, BHK-21 cells were infected with the mutant viruses at a multiplicity of infection of 5 at 37°C, and the parent virus Asia1/YS/CHA/05 was used as a control. After adsorption for one hour, cells were rinsed with phosphate-buffered saline (PBS, pH 7.4) to remove unabsorbed virus and incubated in Dulbecco’s modified Eagle’s medium (DMEM) supplemented with 2% fetal calf serum. Supernatants were harvested after 2, 4, 6, 8, 10, 12 and 14 h of incubation at 37°C and stored at -70°C. Virus titers were determined and expressed as 50% tissue culture infectious doses (TCID_50_) using BHK-21 cells grown in 96-well tissue culture plates according to standard procedures. Five days after infection, the wells exhibiting cytopathetic effect (CPE) were counted as positive, and the virus titer (TCID_50_) was determined. We calculated average values and standard deviations for three independent experiments. In parallel, cell monolayers infected as described before were collected in TRIzol^©^ (Invitrogen) and intracellular RNA was obtained following manufacturer’s instructions; total RNA in each sample was measured using a NanoVue Spectrophotometer. cDNA was synthesized in the presence of AMV reverse transcriptase (TaKaRa) using 0.5 μg of Random Primers (TaKaRa). Finally, a real time-PCR assay was performed in a Light Cycler® 480 Real-Time PCR System (Roche Diagnostics) using a SYBR® Premix Ex TaqTM (TaKaRa) and a standard amplification profile with the primers D1 (AGACTCGCATTGTCGATGTCTTG, forward primer) and D2 (CAAATCTTTGCCAATCAA TATCAG, reverse primer), to obtain a 150 bp fragment from the 3D region of FMDV genome.

### LD_50_ for virulence assay

To evaluate the pathogenicity of the rescued viruses, the mutant viruses and the parent strain Asia1/YS/CHA/05 were inoculated into suckling BALB/c mice. Briefly, 3-day-old of BALB/c suckling mice were divided into 24 groups (4 mice per group) and inoculated cervicodorsally with 200 μL of six serial tenfold dilutions of each virus or PBS. Morbidity of the animals was examined every six hours until seven days after inoculation, and LD_50_ values were calculated by the Kärber method. The experiments were repeated for three times and standard deviations were calculated.

## Abbreviations

FMD: Foot-and-mouth disease; FMDV: Foot-and-mouth disease virus; nMAb: Neutralizing monoclonal antibody; MAb: Monoclonal antibody; IFA: Indirect immunofluorescence assay; VNT: Virus neutralization test; DMSO: Dimethyl sulfoxide; ELISA: Enzyme-linked immunosorbent assay; dNTP: Deoxyribonucleotide triphosphates; NT_50_: Neutralizing titer; PBS: Phosphate-buffered saline; DMEM: Dulbecco’s modified Eagle’s medium; TCID_50_: 50% tissue culture infectious doses; CPE: Cytopathetic effect.

## Competing interests

The authors declare no conflict of interest.

## Authors’ contributions

MX Conceived of the study, participated in its design and coordination, performed research and drafted the manuscript. HW Participated in the study design and in the editing of the manuscript. WL, GZ, YTs participated in the study design and carried out the MAb studies. LY participated in the study design, the manuscript modification and submission. All authors read and approved the final manuscript.
